# A direct observation tool to measure interactions between shade, nature, and children’s physical activity: SOPLAY-SN

**DOI:** 10.1186/s12966-022-01355-4

**Published:** 2022-09-29

**Authors:** Allison Poulos, Kylie Wilson, Kevin Lanza, Jennifer Vanos

**Affiliations:** 1 College of Health Solutions, Arizona State University, Phoenix, AZ USA; 2 School of Public Health, University of Texas Health Science Center at Houston, Houston, TX USA; 3 School of Sustainability, Arizona State University, Tempe, AZ USA

**Keywords:** Children, Physical activity, Shade interaction, Nature interaction, Playgrounds, Recess, Schools

## Abstract

**Background:**

Most physical activity (PA) during school occurs at recess; however, recess PA may be influenced by children’s thermal comfort and interaction with nature, neither of which have concurrently been measured reliably in previous studies. This study tests the reliability of SOPLAY-SN, an adaption of the validated System for Observing Play and Leisure Activity in Youth (SOPLAY) to measure Shade and Nature (SN) alongside PA, and associations between children’s PA and interaction with shade and nature during recess to highlight the utility of the tool.

**Methods:**

Interactions with shade and nature were measured using systematic direct observation at two playgrounds (primary-grade = ages 5–8, upper-grade = ages 9–12) during recess at an elementary school in Phoenix, Arizona (USA). Pairs conducted observations over four warm days (primary = 29–34 °C, upper-grade = 32–36 °C) in May 2021 (*N* = 179 scans). Intraclass correlation coefficients (ICC) were used to calculate inter-rater reliability. Mean counts, frequencies, and Kendall rank correlation coefficient tests were used to assess relations between PA level and interactions with shade and nature.

**Results:**

Reliability was good for sedentary behavior (ICC = 0.98); light PA (LPA; ICC = 0.80) and moderate-to-vigorous PA (MVPA; ICC = 0.94); shade interaction (ICC = 0.95); and nature interaction (ICC = 0.80) and average agreement was good (86% overall PA, 88% shade, 90% nature). Most (60%) primary-grade children were observed in the shade, with 64% under a covered play structure where children were mainly (47%) sedentary. Of the 11% of primary-grade students observed interacting with nature, 90% occurred in a grass field with trees. Among upper-grade children, 23% were observed in the shade with 53% in grass fields where 48% of play was light. Few (7%) upper-grade children were observed interacting with nature, with most instances (76%) in a grass field with trees. Among primary-grade children, shade was correlated with sedentary behavior (*τ*_b_ = 0.63, *p* < .05); LPA (*τ*_b_ = 0.39, *p* < .05); MVPA (*τ*_b_ = 0.56, *p* < .05); and nature interactions with sedentary behavior (*τ*_b_ = 0.16, *p* < .05). Among upper-grade children, shade was correlated with sedentary behavior (*τ*_b_ = 0.27, *p* < .05) and LPA (*τ*_b_ = 0.21, *p* < .05).

**Conclusions:**

SOPLAY-SN is a reliable tool for measuring children’s interaction with shade and nature and participation in PA. Understanding how shade and nature impact movement during recess can inform playground design for children’s health and well-being.

**Supplementary Information:**

The online version contains supplementary material available at 10.1186/s12966-022-01355-4.

## Background

Engaging in regular physical activity (PA) and limiting sedentary behavior during childhood are independently associated with important health benefits, such as reduced risk of childhood obesity [1, 2] and associated chronic diseases (e.g., type 2 diabetes, certain cancers) [3–6], yet less than one-third of children ages 6–17 years are currently meeting PA or sedentary time recommendations [7]. Schools are important for children’s PA as they provide access, structure, and systems to support healthy behaviors and health behavior change [8] and are one of the few settings that reach nearly all children [9, 10], with most children spending almost half of their waking hours at school.

Physical education, recess, classroom PA breaks, and before- and after-school programs all contribute to PA accrual of elementary students [11, 12]. Of these, recess provides up to 44% of all school-based PA [13] and counters the six to eight hours of sedentary time children typically incur during waking hours [5, 14–16]. However, children’s engagement in PA during recess is contingent upon multiple factors including the design of play spaces [17] and the availability of equipment [18–21]. Additionally, a growing body of research has identified that thermal comfort—especially during extreme cold or heat conditions [22–24]—and the amount of nature or greenspace [25] are important factors related to children’s PA in schoolyards. Previous studies have modeled physiological equivalent temperature (i.e., thermal index derived from the human energy balance) in schoolyards to estimate that children may be more thermally comfortable in areas covered by shade, especially from trees, compared to areas exposed to the sun [26–28]. Another study used the comfort formula (COMFA) energy budget model to assess children’s thermal comfort during PA, finding children to be more comfortable engaging in PA in shaded compared to unshaded playgrounds [24]. These studies assessed the relations between shade and thermal comfort of children in schoolyards, yet do not measure children’s behaviors of seeking shade and nature, both of which may provide thermal comfort and promote PA in temperature extremes. There is a need for a simple and comprehensive tool to assess the interactions of children with shade and nature in concert with PA.

PA intensity during recess may be modified by individuals’ heat exposure and associated level of thermal comfort. With climate-related factors becoming increasingly recognized as drivers of PA [29–31], incorporating measures of heat exposure and shade during recess is particularly relevant in warm climates. For instance, in the humid subtropical climate of Austin, Texas, children were more likely to be observed under tree canopy during high ambient temperatures compared to lower temperatures, potentially seeking shade for thermal comfort [32]. Further, children’s PA levels during recess have been found to be lower during the spring and summer in Melbourne, Australia [33], which may be attributable to thermal discomfort during these warmer seasons in the temperate oceanic climate.

Interacting with nature has been shown to improve the physical health, psychological health, and well-being of youth [34]. Associations between nature, PA, and sedentary behavior exist among children [35] and adolescents [36]. For example, time spent outdoors by children is positively associated with moderate-to-vigorous physical activity (MVPA) and negatively associated with sedentary behavior [37–40]. A commonly referenced theoretical underpinning for the observed increases in PA levels and intensities while in nature or greenspace is the Biophilia hypothesis [41], which suggests human’s biological affinity for and desire to connect with nature as the basis for the improvements in health associated with nature exposure. Further, vegetation and water bodies cool the nearby air and surfaces, as does shade from trees [42]. Nature on school playgrounds may serve as a solution to thermal comfort via air temperature and radiation reduction [43] that can help to promote PA and provide other benefits to health and well-being.

To our knowledge, there is no research instrument for PA measurement that also reliably measures individuals’ interactions with shade and nature. Researchers typically use three types of PA measurement techniques in recess settings: self-report, device-based measurement, and direct observation. Self-report has traditionally occurred through surveys, recalls, or interviews [44]. While generally a low-cost and feasible strategy, particularly with larger sample sizes, collecting information through self-report is associated with response bias. Children have trouble recalling PA behavior [45] and often overestimate PA levels [46]. Self-reported accounts of time in shade and nature are susceptible to the same likelihood of recall bias [47], but also associated with social desirability bias found in environmental research, the tendency of individuals to respond more favorably to present a better social impression [48]. Further, while self-report PA measures provide valuable information about the types and the contexts of PA children engage in, they are less helpful for assessing other relevant characteristics such as duration and intensity [49] that are particularly salient in developing PA recommendations.

In light of the shortcomings of PA measurement via self-report, device-based PA measures (e.g., heart rate monitors, pedometers, accelerometers) have gained popularity for producing reliable, valid, and stable results in both adults and children [50]. PA measurement devices are considered minimally invasive and can record various movement characteristics over multiple days; however, devices are costly and using them often requires knowledge and access to software that can be difficult to navigate [51]. Although the effects of seasonality may be accounted for in analyses, device-based PA instruments alone cannot currently capture interaction with shade or nature. Researchers have quantified shade using the sky view factor (i.e., fraction of visible sky) [52, 53], or the amount of greenness and tree canopy via satellite-based Normalized Difference Vegetation Index [54] and classification of high-resolution aerial imagery, respectively [55, 56]; however, these methods have not counted the number of individuals under shade versus unshaded for a given area. Determining individuals’ interaction with nature by linking geolocated PA measured by participant-worn accelerometers and GPS devices with geolocated nature is resource-intensive and requires specialized training [32].

Systematic direct observation is a structured process that uses visual scans to measure group behavior and is advantageous for its strong internal validity and ability to assess both the physical and social environment [57], two major influences on PA during recess [58]. Direct observation is a less costly and more accessible method for assessing both physical and social contexts of PA among large samples than combining device-based PA measurement with self-report [51, 57]. In addition, direct observation reduces participant burden and reactivity due to limited interaction between observers and participants. Despite an abundance of research using direct observation to study PA at recess, the mediating factors that may explain children’s PA—including interactions with shade and nature/greenspace [16, 25, 59]—are not well understood during this critical time for PA accrual [57]. Researchers have quantified shade [60, 61] and the amount of nature [62, 63] using direct observation, but these instruments are not designed to measure the number of individuals under shade versus unshaded for identified areas and have not been assessed for reliability [32, 64]. The Tool for Observing Play Outdoors (TOPO) by Loebach and Cox [65] is an outdoor field observational protocol that categorizes children’s activities in outdoor and naturalized play spaces to evaluate outdoor play behaviors and the environment. The authors created “bio play” and "restorative” categories for outdoor play types, but there is no emphasis on shade interaction.

To better understand how shade and nature influence children’s PA during recess, there is a need for a simple and comprehensive tool. Conceptualized under the Youth Physical Activity Time, How, and Setting (Y-PATHS) framework, which recognizes the timing, type, and setting influences the PA behavior of children and adolescents [66], we extend the application of the System for Observing Play and Leisure Activity in Youth (SOPLAY)—a validated instrument of systematic direct observation used extensively to measure group levels of PA during recess [67]—by adapting the tool to include interactions with shade and nature to further explain why children move as they do during recess in relation to the location and presence of shade and nature. We test the reliability of our newly developed observational tool, SOPLAY-SN, during school-based outdoor free play and explore associations between PA, shade, and nature interaction.

## Methods

### Research design and setting

The study consisted of two phases: 1) instrument development and pilot testing and 2) site observations. We present herein baseline results from site observations. To test the reliability of SOPLAY-SN in both phases, we used a partially crossed design where we independently observed subsets of child participants by different pairs of trained observers to assess the consistency and degree of agreement between coded observation characteristics.

**Fig. 1:**
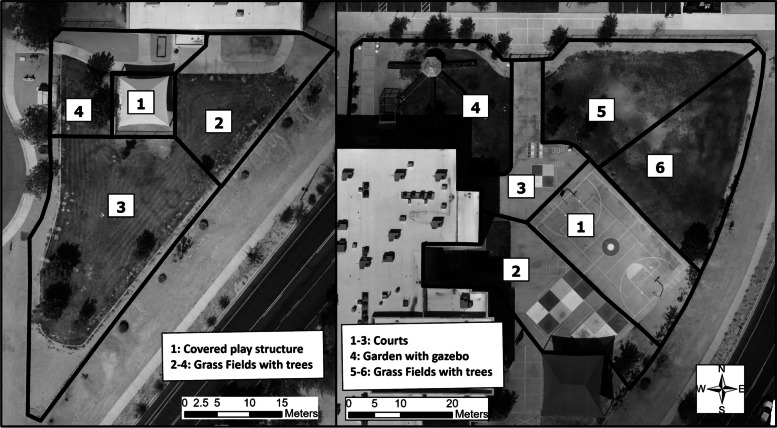
Target areas for systematic direct observation at the primary-grade playground (left) and upper-grade playground (right)

Participants included primary-grade children (age 5–8 years) and upper-grade children (age 9–12 years) at one public elementary school in Phoenix, AZ. The school and participants were already enrolled in a larger, longitudinal study assessing associations between the school environment and children's health. The school enrolled 773 students in the 2020–2021 academic year with 53% female, 67% Hispanic, and 72% low income (qualifying for free or reduced-price meals), [68] slightly higher than the percentages of school children in the greater Phoenix Metropolitan Region (48% female, 43% Hispanic, 49% low income [69]). Ethical approval was granted by Arizona State University (Study #00010114). The school principal provided consent with the option for parents and guardians to opt out of participation at any time. Observations occurred at two outdoor areas on school grounds (Fig. 1): 1) a primary playground (1,524 square meters) with a shade structure and a large grass field with trees, and 2) an upper-grade playground (3,428 square meters) with courts, a large grass field with trees, a garden, and no shade structures.

### Phase I—Instrument development and pilot testing

#### SOPLAY adaptation

We adapted SOPLAY to include measures of shade and nature interactions using the same momentary time sampling procedures as in the original instrument [70]. Shade interaction was measured by recording the number of children in partial or total shade. The source of the shade could be natural (i.e., trees) or artificial (i.e., shade structure, building). Nature interaction was classified as either tactile (e.g., climbing trees, picking flowers, playing in the dirt) or verbal (e.g., talking about hopping from rock to rock, deciding on trees as play boundaries). These criteria were developed by the research team to be used broadly in school playground settings and were based on other adaptations of direct observation tools used in joint-use parks [32] and early childhood centers [64].

#### Observer training

Six observers were trained for 20 total hours over three days using standard SOPLAY training materials. The observers were initially trained using online videos by SOPLAY developers and then progressed to short, videotaped recess segments for practice until the team met an acceptable observer agreement of greater than 80% [46]. Reliability was assessed by evaluating agreement in coding the final assessment included in the training videos from criteria provided by the original SOPLAY developer [70].

#### Data collection

We pilot tested the adapted SOPLAY instrument using approximately 34 min of video footage (ranging 2–12 min in length) of four primary recess periods collected over three days. Observational scans were recorded 12 times by four trained observers who coded independently. During each video, visual scans were conducted at five-minute intervals beginning with girls’ PA, then boys’ PA, followed by shade and nature interactions for both sexes. Each scan began when the video started, with observers recording information between scans. Observers scanned and recorded the number of girls and boys being sedentary, engaging in LPA, and engaging in MVPA, and then scanned and recorded the number of girls and boys in the shade and interacting with nature using momentary time sampling [57].

### Phase II—Site observations

#### Data collection

Observations were conducted at the primary- and upper-grade playgrounds during four recess periods over a span of four days in May 2021. Observations began at the start of each recess and lasted the duration of the 15-min recess period. Between three to six observers were present for each recess observation. Air temperatures ranged 29–34 °C during recess periods at the primary playground (10:00–10:15am and 11:45am–12:00 pm) and 32–36 °C (12:10–12:25 pm and 1:50–2:05 pm) at the upper-grade playground. These data were collected from Arable, Inc. weather stations situated within each play area. Temperatures recorded are similar to average regional temperatures in May (mean = 28 °C, minimum = 21 °C, maximum = 35 °C) [71].

Prior to data collection, a map of the playground was divided into target areas to represent locations likely to provide children with opportunities for PA. The primary playground contained four target areas and the upper grade playground contained six target areas (Fig. 1). In the primary playground, Target Area 1 (42% shade, 45% nature) was beneath a covered play structure with wood chip bedding. Target Areas 2–4 (15% shade, 94% nature) were grassy fields with some trees. In the upper grade playground, Target Areas 1–3 (2% shade, 16% nature) were courts with various markings for play (e.g., four square, basketball) and permanent basketball hoops. Some small trees lined the perimeter of the playground fence in these areas. Target Area 4 (10% shade, 98% nature) was a garden with grass and small shrubs. Target Areas 5 and 6 (5% shade, 100% nature) were large grassy fields with a few trees and shrubs along the perimeter of the fencing. Permanent soccer goals were also present. Nature was calculated to represent any combination of grass, gravel, rocks, trees, bushes, gardens, and/or dirt.

All target areas were observed in a specific order for each recess period. Observers stood on the periphery of target areas to reduce the likelihood of reactivity (i.e., the chance that children would change behavior if aware of being observed) [57]. Observers conducted visual scans from left to right at an approximate rate of one child per second, counting the number of children being sedentary (i.e., sitting or standing) and engaging in LPA (i.e., walking or slow-moving activities) and MVPA (i.e., running, climbing, or fast-paced games or sports). This process began with successive scans for PA, shade, and nature interactions for girls, and then for boys. Environmental factors were also recorded for each target area including accessibility, usability, presence of supervision, presence and type of organized activity, and equipment availability. Additionally, observers recorded the time of day, length of recess, and weather conditions. In-person observations were recorded on paper using the standardized SOPLAY instrument with an additional two shade and nature categories (Additional file 1: Appendix) using a clicker mounted on a clipboard to record behavior [67].

Pairs of observers, standing approximately 4.5 m (i.e., 15 feet) apart to ensure no information was shared during observations, scanned independently to test inter-rater reliability [57]. One researcher was also designated as the criterion and reliability coder and collected SOPLAY data throughout the study. Inter-rater reliability testing was conducted during pilot testing and site visits by rotating pairs of observers to calculate agreement between four different pairs of observers.

### Data analysis

Intraclass correlation coefficients (ICC) using a one-way average measures random effects model were used to calculate inter-rater reliability. Interobserver ICCs for PA, shade, and nature were calculated to represent the overall consistency across all combined observations.

Mean observation counts were calculated for each target area and for the overall playground, separately for boys and girls. For observations performed as reliability checks, averages of the two simultaneous observations were used. Vigorous levels of physical activity were determined to represent MVPA, per SOPLAY design [70]. A series of Kendall rank correlation coefficient tests (Kendall’s tau-b) were used to assess associations between PA level, and interactions with shade and nature by playground and sex of child.

## Results

### Reliability

The final observer trainings in Phase I included 15 scans with varying difficulty (e.g., different environmental contexts) and numbers of participants (3–12 individuals per scan). ICC ranged 0.80–0.90 indicating good reliability [72]. A total of 48 scans were conducted during pilot testing (N_Phase I_ = 68). The reliability measured between observers during pilot testing in Phase I was acceptable (Table 1) [72]. ICC estimates ranged from 0.55 (95%CI 0.24–0.76) to 0.92 (95%CI 0.84–0.95) and average agreement was good with 91% for overall PA, 81% for shade, and 82% for nature (agreement data not shown).

**Table 1:** Intraclass agreement calculations between observer pairs and physical activity, shade, and nature observations

	**Intraclass Correlation Coefficient**	**95% Confidence Interval**
**Phase I- Between Observers (**48 scans; N_Phase I_ = 68)		
Pair 1	0.59	0.37–0.74
Pair 2	0.92	0.84–0.95
Pair 3	0.71	0.54–0.82
Pair 4	0.55	0.24–0.76
**Phase II—Between Observers (**179 scans; N_Phase II_ = 212)		
Pair 1	0.88	0.37–0.99
Pair 2	0.90	0.43–0.99
Pair 3	0.94	0.64–0.99
Pair 4	0.98	0.87–0.99
**Phase II—Between physical activity, shade, and nature observations**		
Sedentary	0.98	0.89–0.99
Light PA	0.80	0.21–0.97
Moderate-to-Vigorous PA	0.94	0.68–0.99
Shade	0.95	0.71–0.99
Nature	0.80	0.20–0.97

A total of 179 scans were conducted during Phase II (N_Phase II_ = 212). Reliability during site visits in Phase II was good (greater than 0.80). For overall inter-rater reliability, ICCs between four pairs of observers ranged from 0.88 (95%CI 0.37–0.99) to 0.98 (95%CI 0.87–0.99). Average agreement was 86% for overall PA, 88% for shade, and 90% for nature. ICCs were investigated for sedentary behavior (ICC 0.98, 95%CI 0.89–0.99); LPA (ICC 0.80, 95%CI 0.21–0.97); MVPA (ICC 0.94, 95%CI 0.68–0.99); shade (ICC 0.95, 95%CI 0.71–0.99); and nature (ICC 0.80, 95%CI 0.20–0.97).

### Physical activity, shade, and nature

#### Playground use of primary-grade children

On average, almost half (41%) of primary-grade children were observed engaging in sedentary behavior (Table 2). The majority (55%) of primary-grade children were observed in grass fields (Target Areas 2–4). Most children were sedentary when under the covered play structure area (47%) and in grass fields (37%). Girls were most frequently observed as sedentary (48%), while boys were most frequently observed engaging in MVPA (41%). Girls were most frequently observed in the covered play structure area (53%), wherein most were sedentary (51%). Boys were most frequently observed in grass fields (65%), wherein most were engaging in MVPA (41%).

**Table 2:** Average percentage of time in PA levels, shade, and nature overall for primary-grade girls and boys **(**179 scans; N_Phase II_ = 212)

	**Physical Activity**				**Shade**		**Nature**	
	**Total Observations**	**Sedentary**	**LPA**	**MVPA**	**Total Observations**	**Shade**	**Total Observations**	**Nature**
	**(%)**	**(%)**	**(%)**	**(%)**	**(%)**	**(%)**	**(%)**	**(%)**
**All Students**								
Across Entire Playground	-	41	26	33	60	-	11	-
Within Target Areas								
Covered Play Structure	45	47	21	32	38	64	1	10
Grass Fields with Trees	55	37	30	33	22	36	10	90
**Girls**								
Across Entire Playground	-	48	24	28	65	-	9	-
Within Target Areas								
Covered Play Structure	53	51	19	30	48	74	2	19
Grass Fields with Trees	47	46	28	26	17	27	7	81
**Boys**								
Across Entire Playground	-	31	28	41	54	-	12	-
Within Target Areas								
Covered Play Structure	35	40	23	37	28	52	0	2
Grass Fields with Trees	65	27	31	41	26	48	12	98

Note: Covered play structure = Target Area 1. Grass fields with trees = Target Areas 2–4. *LPA* Light physical activity, *MVPA* Moderate-to-vigorous physical activity

Approximately 60% of primary-grade children were observed in the shade, with the majority (64%) of these observations occurring under the covered play structure. Among girls, 65% were observed in the shade with 74% of these observations occurring under the covered play structure. Just over half (54%) of the boys at recess were observed in the shade, with most (52%) of those observations also occurring under the covered play structure.

On average, 11% of the primary-grade children were observed interacting with nature with the majority (90%) of these observations occurring in grass fields. About 9% of girls were observed interacting with nature with the majority (81%) of these observations occurring in grass fields. Compared to girls, boys interacted with nature at a slightly higher frequency (12%), primarily in grass fields (98%).

##### Playground associations of primary-grade children

**Fig. 2:**
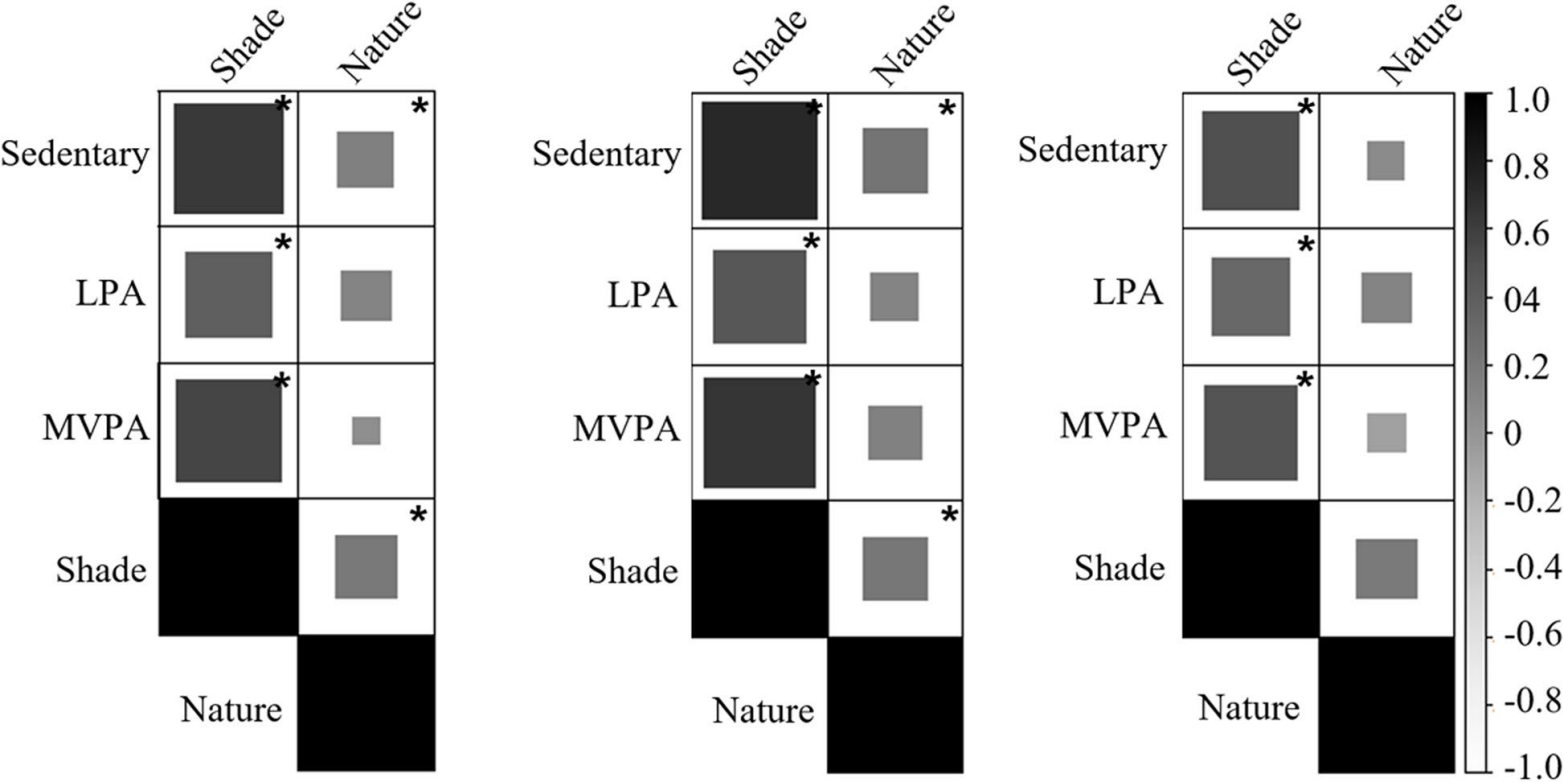
Kendall’s tau-b correlations between physical activity level, and shade and nature interactions for all children (left), girls (middle), and boys (right) in primary grade. *Correlation significant at alpha = .05 level. LPA = Light physical activity. MVPA = Moderate-to-vigorous physical activity

Among all primary-grade children, we found moderate positive associations between shade and sedentary behavior (*τ*_b_ = 0.63, *p* < 0.05); LPA (*τ*_b_ = 0.39, *p* < 0.05); and MVPA (*τ*_b_ = 0.56, *p* < 0.05) (Fig. 2). There was also a weak positive association between nature interactions and sedentary behavior (*τ*_b_ = 0.16, *p* < 0.05) and shade (*τ*_b_ = 0.20, p < 0.05). Among girls, we found similar moderate associations between shade and LPA (*τ*_b_ = 0.45*, p* < 0.05) and MVPA (*τ*_b_ = 0.65, *p* < 0.05), and a strong correlation between sedentary behavior and shade (*τ*_b_ = 0.72, *p* < 0.05). We found similar weak associations between nature interaction and sedentary behavior (*τ*_b_ = 0.23, *p* < 0.05) and shade (*τ*_b_ = 0.21, p < 0.05) among observations of girls. Among boys, shade was moderately associated with sedentary (*τ*_b_ = 0.50, *p* < 0.05), LPA (*τ*_b_ = 0.32, *p* < 0.05) and MVPA (*τ*_b_ = 0.47, *p* < 0.05), but nature was not correlated with either shade or PA.

##### Playground use of upper-grade children

Most of the upper-grade children were observed engaging in sedentary behavior (45%) or LPA (42%) (Table 3). Girls were most frequently observed engaging in sedentary behavior (49%), while boys were most often observed engaging in LPA (42%). The majority (61%) of children were observed playing on the courts (Target Areas 1–3), wherein most students were sedentary (girls = 50%, boys = 47%).

**Table 3:** Average percentage of time in PA levels, shade, and nature overall for upper-grade girls and boys **(**179 scans; N_Phase II_ = 212)

	**Physical Activity**				**Shade**		**Nature**	
	**Total Observations**	**Sedentary**	**LPA**	**MVPA**	**Total Observations**	**Shade**	**Total Observations**	**Nature**
	**(%)**	**(%)**	**(%)**	**(%)**	**(%)**	**(%)**	**(%)**	**(%)**
**All Students**								
Across Entire Playground	-	45	42	13	23	-	7	-
Within Target Areas								
Courts	61	49	38	13	8	36	1	12
Garden	4	45	42	13	3	11	1	12
Grass Fields with Trees	35	38	48	14	12	53	5	76
**Girls**								
Across Entire Playground	-	49	42	8	23	-	6	-
Within Target Areas								
Courts	59	50	41	9	10	42	1	23
Garden	4	27	68	5	2	11	1	11
Grass Fields with Trees	37	50	41	9	11	47	4	66
**Boys**								
Across Entire Playground	-	41	42	17	24	-	9	-
Within Target Areas								
Courts	63	47	36	17	6	26	0	1
Garden	4	62	19	19	3	12	1	14
Grass Fields with Trees	33	26	56	18	15	62	8	85

Note: Courts = Target Areas 1–3. Garden = Target Area 4. Grass Fields with Trees = Target Areas 5–6. *LPA* Light Physical Activity, *MVPA* Moderate-to-vigorous physical activity

Approximately 23% of upper-grade children were observed under the shade, with the majority (53%) in grass fields with some trees (Target Areas 5–6). Among girls, 23% were observed in the shade with the majority (47%) of those observations occurring in grass fields with trees. Approximately one-quarter (24%) of boys were observed in the shade, with most (62%) of these observations also occurring in grass fields with trees.

Additionally, an average of 7% of upper-grade children at recess were observed interacting with nature, mainly in grass fields (76%). An average of 6% of girls and 9% of boys were observed interacting with nature with the majority (66% and 85%, respectively) of these observations occurring in grass fields.

### Playground associations of upper-grade children

**Fig. 3:**
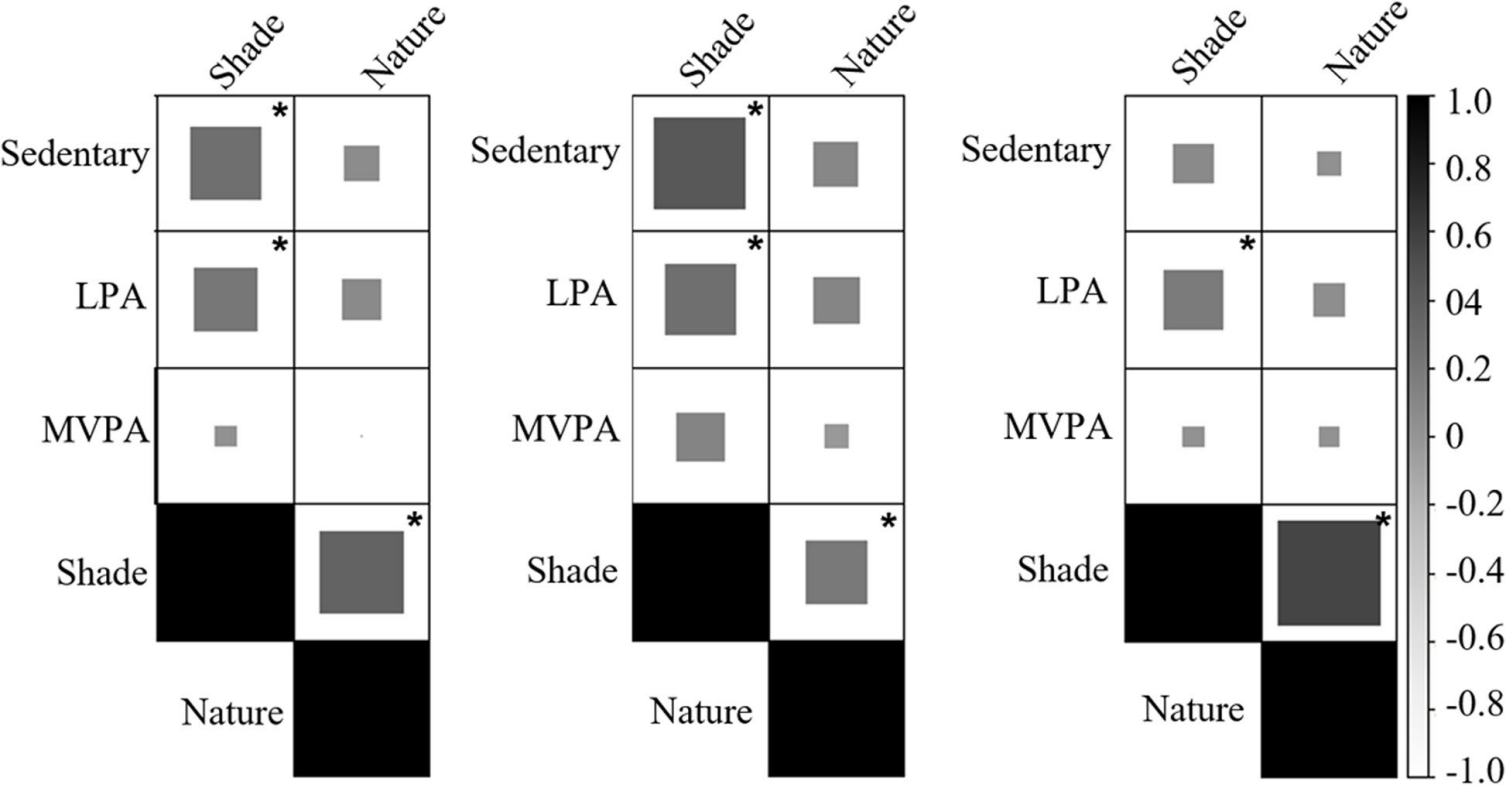
Kendall’s tau-b correlations between physical activity level, and shade and nature interactions for all children (left), girls (middle), and boys (right) in upper grade. *Correlation significant at alpha = .05 level. LPA = Light physical activity. MVPA = Moderate-to-vigorous physical activity

Among upper-grade children, associations were positive and weak, including shade and sedentary behavior (*τ*_b_ = 0.27, *p* < 0.05); LPA (*τ*_b_ = 0.21, *p* < 0.05); and nature interactions (*τ*_b_ = 0.36, p < 0.05) (Fig. 3). No other associations were statistically significant for upper-grade children (p > 0.05). Among girls, shade was moderately associated with sedentary behavior (*τ*_b_ = 0.44, *p* < 0.05) and weakly associated with LPA (*τ*_b_ = 0.27*, p* < 0.05) and nature interactions (*τ*_b_ = 0.20, *p* < 0.05). Among boys, shade was weakly associated with LPA (*τ*_b_ = 0.18, *p* < 0.05) and moderately associated with nature interactions (*τ*_b_ = 0.56, *p* < 0.05).

## Discussion

Measuring children’s physical activity during recess is critical as the majority of school-based PA occurs during recess time [13, 73, 74]. Children’s activity levels are influenced by shade and nature interactions, neither of which have been measured concurrently with PA during recess. Herein, we developed a reliable tool for measuring children’s PA and interactions with shade and nature, and presented associations to demonstrate its application. SOPLAY-SN captures objective information on these behaviors with strong internal (face) validity, a characteristic of direct observation instruments [57]. SOPLAY-SN is an improvement over contemporary methods because it measures three behaviors (i.e., PA, shade interaction, nature interaction) in a single instrument, minimal training, ease of implementation, and low cost. There is opportunity to utilize SOPLAY-SN for measuring other PA domains of children in addition to free play (i.e., transportation, functional, organized activities); other times of day (i.e., school days out of school time, non-school days); and settings outside of the school environment (e.g., natural areas, outdoor recreational facilities, residential spaces) [66].

SOPLAY-SN can be a useful tool for public health surveillance and for school- and community-based health practitioners when designing public spaces as it provides group-level PA estimates situated within contextual settings. For instance, SOPLAY-SN can be used to test the effectiveness of design interventions for promoting safe PA of children in high-temperature conditions. The current study performed on warm days showed children seeking shade for rest (little-to-no PA). Adding shade could provide a cooler schoolyard microclimate with greater sun and heat protection during MVPA, not only during rest. With climate change causing an increase in overall temperatures and in the intensity, frequency, and duration of heat waves [75], it is necessary to create, test, and implement new strategies that will reduce heat loads on children during outdoor play [76]. The use of SOPLAY-SN in testing such designs has value in a range of climates. In warm climates, individuals are currently adapting to high temperatures and will have to further adjust to projected temperature increases from climate change and urban growth [75]. In cooler climates, individuals are less acclimatized to heat and have fewer heat adaptation practices in place (e.g., adopting preventative outdoor behaviors); thus, they may be more vulnerable to projected increases in extreme heat events [77, 78]. Environmental interventions that include nature-based solutions at schoolyards can provide shade and other associated benefits (e.g., flood mitigation, surface cooling, biodiversity) to children’s health and well-being. Strategic planning for high-quality shade types should become a focal point considered throughout the design process for school playgrounds. Programmatic interventions may include changing the scheduling of recess to times of day that are potentially more thermally comfortable (i.e., not peak temperatures during the warm season) to assess the impact on PA levels, shade interaction, and nature interaction.

### Time spent in physical activity levels, shade, and nature

Differences in time spent in shade and interacting with nature existed between primary-grade children (age 5–8 years) and upper-grade children (age 9–12 years), with younger children spending more time in shaded areas and interacting with nature. More interaction with nature may be due to a higher percentage of space with nature in the primary play space, as well as a smaller overall area of play. Fjortoft et al. found that greener, more attractive play spaces in Norway were associated with higher levels of PA [79]. Similarly, a study by Wheeler et al. used personal location and heart rate monitoring for tracking children’s PA outdoors in the U.K., finding that children’s activity levels were higher when playing in greenspaces, whether shaded or not [40]. Simultaneously monitoring location via global positioning system devices and personal heart rate is also valuable for indicating locations of high or low PA levels [80].

### Associations between physical activity, shade, and nature

In both primary- and upper-grade observations, shade was positively correlated with sedentary behavior and LPA. Most (47%) of the primary children who were sedentary were observed under a covered play structure, with shade use in this age group having the highest correlation with sedentary behavior. These results align with those from Vanos et al., which found that children of similar age spent their time resting in the shade and actively playing in the sun-lit areas on hot sunny days in California [81]. However, a study by Dobbinson et al. in Australia found that children’s activity was not hindered by implementing a shade sail. Children utilized the shaded area more frequently during their outdoor play [82]. A study in Texas by Vanos et al. found that on hot, sunny days, children were more thermally comfortable in the shade, and solar radiation and air temperature were significant predictors of thermal comfort [24]; hence, if children are warm during play, they may seek shade for rest and cooling.

Few studies have examined associations between PA and weather or playground microclimates among children [83]. Some evidence supports the effect of rainfall on decreased PA and increased sedentary time among children during the school day [83]. However, improved understanding is needed surrounding the role of the play space design on outdoor thermal comfort, sun exposure, movement, and overall heat load (i.e., air temperature, relative humidity, airflow, radiation, clothing, PA) to support safe play and learning.

### Limitations

While the utility of SOPLAY-SN to comprehensively measure group levels of PA along with shade and nature interactions is a strength, we note limitations of the instrument. SOPLAY-SN scans were performed in succession, with observations of PA followed by shade, then nature. Because each scan lasted approximately 10 s, there was a slight lag between observations in each category. Utilizing this protocol allowed for greater accuracy among observers, but further research is needed to test the reliability of successive scans compared to scans of PA, shade, and nature at exact moments in time. Future studies should also consider shade observations as either natural or artificial to inform future playground design.

Regarding study design, we acknowledge that our focus on a single school in the Southwestern United States limits the generalizability of our findings. Future studies should use SOPLAY-SN in spaces in other regions with different, but comparable, PA infrastructure and levels of shade and nature to test differences in behavior based on these elements (e.g., play structure with and without shade covering). We were also unable to conclude whether behavioral differences between primary- and upper-grade children were driven by age or differences in playground designs. Future research can examine variability in PA, shade, and nature associations by age among children playing in the same recess space. Finally, future studies can expand on the impact of air temperature on behavior. In our study, all observations were conducted during days with relatively high temperatures, preventing the comparison of results between high-, moderate-, and low-temperature days.

## Conclusions

The results of this study support the reliability of SOPLAY-SN as a tool to measure group levels of physical activity and interactions with shade and nature among children at recess. We found differences in time spent in shade and interacting with nature between primary-grade children (age 5–8 years) and upper-grade children (age 9–12), with younger children spending more time in shaded areas and interacting with nature. Shade was positively correlated with both sedentary behavior and light physical activity among both primary- and upper-grade children. Identifying determinants of children’s physical and sedentary activities has implications for the design of the built environment for population health improvement. SOPLAY-SN can be a useful tool for public health surveillance and to inform the design of both school and community play spaces, and should be utilized in other settings with varying playground designs and degrees of shade and nature.

## Supplementary Information

**Additional file 1.** Appendix.

## Data Availability

The dataset supporting the conclusions of this article is available at https://www.openicpsr.org/openicpsr/project/169661/.

## Abbreviations

PA: Physical activity
SOPLAY-SN: System for Observing Play and Leisure Activity in Youth-Shade and Nature
ICC: Intraclass correlation coefficients
LPA: Light physical activity
MVPA: Moderate-to-vigorous physical activity
