# Supplementary material for: A direct observation tool to measure interactions between shade, nature, and children’s physical activity: SOPLAY-SN
Source: Int J Behav Nutr Phys Act. 2022 Sep 29;19:127. doi: 10.1186/s12966-022-01355-4 (PMC9524068; doi:10.1186/s12966-022-01355-4)

**Appendix**

CONDITION

A = Accessible (e.g., not locked)

U = Usable for physical activity (e.g., not excessively wet)

S = Supervised by school personnel (e.g., teachers) (supervisor must be in or adjacent to target area (i.e., available respond to emergencies), but does not have to be instructing or organizing activities

O = Organized physical activity

E = Equipment provided by school is present (e.g., balls, jump ropes). Do not code 'YES' if the only equipment is permanent (e.g., basketball hoops)

| School ID : ___ ___  Date:__ __/__ __/__ __ |
| --- |

# **SOPLAY-SN**

Obs. Init: ___ ___ Reliability: N Y Temp: ___ ___ ^S = Sedentary; L = Light PA; V = Vigorous or Moderate-to-Vigorous PA^

| START TIME | TARGET AREA | CONDITION | | | | | | | Physical Activity | | | | | | | Shade | Nature |
| --- | --- | --- | --- | --- | --- | --- | --- | --- | --- | --- | --- | --- | --- | --- | --- | --- | --- |
|  |  | A | U | S | O | E | | S | | L | | V | | Activity | |  |  |
| __ __: __ __ | _____ | N Y | N Y | N Y | N Y | | N Y | | _____ | | _____ | | _____ | | _____ | _____ | _____ |
|  | _____ | N Y | N Y | N Y | N Y | | N Y | | _____ | | _____ | | _____ | | _____ | _____ | _____ |
| __ __: __ __ | _____ | N Y | N Y | N Y | N Y | | N Y | | _____ | | _____ | | _____ | | _____ | _____ | _____ |
|  | _____ | N Y | N Y | N Y | N Y | | N Y | | _____ | | _____ | | _____ | | _____ | _____ | _____ |
| __ __: __ __ | _____ | N Y | N Y | N Y | N Y | | N Y | | _____ | | _____ | | _____ | | _____ | _____ | _____ |
|  | _____ | N Y | N Y | N Y | N Y | | N Y | | _____ | | _____ | | _____ | | _____ | _____ | _____ |
| __ __: __ __ | _____ | N Y | N Y | N Y | N Y | | N Y | | _____ | | _____ | | _____ | | _____ | _____ | _____ |
|  | _____ | N Y | N Y | N Y | N Y | | N Y | | _____ | | _____ | | _____ | | _____ | _____ | _____ |
| __ __: __ __ | _____ | N Y | N Y | N Y | N Y | | N Y | | _____ | | _____ | | _____ | | _____ | _____ | _____ |
|  | _____ | N Y | N Y | N Y | N Y | | N Y | | _____ | | _____ | | _____ | | _____ | _____ | _____ |
| __ __: __ __ | _____ | N Y | N Y | N Y | N Y | | N Y | | _____ | | _____ | | _____ | | _____ | _____ | _____ |
|  | _____ | N Y | N Y | N Y | N Y | | N Y | | _____ | | _____ | | _____ | | _____ | _____ | _____ |


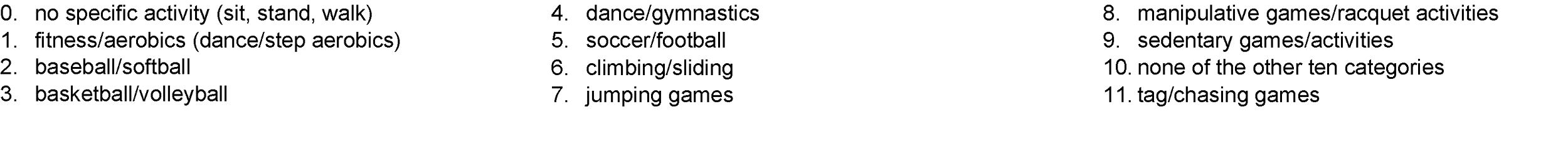

Supplement: Supplementary file 1 — Additional file 1. Appendix. [file 12966_2022_1355_MOESM1_ESM.docx]
